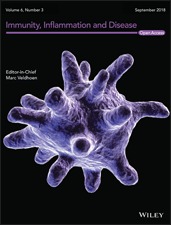# Issue Information

**DOI:** 10.1002/iid3.195

**Published:** 2018-08-29

## Abstract